# Pituitary-immune bidirectional crosstalk under systemic inflammation

**DOI:** 10.1371/journal.pbio.3002440

**Published:** 2023-12-19

**Authors:** Qingyun Li

**Affiliations:** Department of Neuroscience, Department of Genetics, Hope Center for Neurological Disorders, Center for Brain Immunology and Glia, Washington University in St. Louis School of Medicine, St. Louis, Missouri, United States of America

## Abstract

The pituitary gland responds to and modulates immune stress through the hypothalamus–pituitary–adrenal axis. A new study in *PLOS Biology* reveals unconventional bidirectional communication between hormone-producing cells and the immune system upon systemic inflammation.

The pituitary, situated at the hub of the neuroendocrine system, produces distinct hormones in response to neuronal activation in the hypothalamus, thereby modulating the secretory activities of peripheral glands, such as the generation of glucocorticoids by the adrenal cortex [1]. This constitutes the hypothalamus–pituitary–adrenal (HPA) axis, which plays a pivotal role in regulating diverse physiological functions as well as immune responses under homeostasis and stress conditions. It is well-established that peripheral immune challenges may activate the HPA axis via the vagus nerve and the paraventricular nucleus in the hypothalamus, resulting in the secretion of adrenocorticotrophic hormone (ACTH) by corticotropes (one type of hormone-producing cells in the pituitary) and eventual suppression of inflammation [2,3]. However, the transcriptomic changes of pituitary cells in response to immune stimuli remain unknown. In addition, modes of interactions between the pituitary and immune cells under systemic inflammation have not been extensively explored beyond the classical hormonal regulation along the HPA axis.

In this issue of *PLOS Biology*, Yan and colleagues performed bulk and single-cell RNA sequencing (scRNA-seq) analyses on pituitary tissues from mouse models of systemic inflammation to define gene expression changes of hormone-producing cells (HPCs) [4]. They primarily focused on the lipopolysaccharide (LPS) injection paradigm, a robust model for mimicking bacterial infection. scRNA-seq revealed all 6 major HPC types, namely somatotropes, corticotropes, lactotropes, gonadotropes, thyrotropes, and melanotropes, the proportions of which were similarly represented in control and inflammation models (**Fig 1**). Through differential gene expression analyses, the authors identified both stable markers and transcriptional changes for each HPC population upon LPS stimulation. These changes split each HPC group into “healthy” and “inflammatory” states. Differentially expressed genes (DEGs) from HPCs in the single-cell dataset highly correlated with genes identified in the bulk RNA-seq analysis of the entire pituitary tissue, suggesting HPCs as the driving cellular source for the inflammatory response in the pituitary gland. Interestingly, up-regulation of certain genes and pathways, such as members of the *Irf*/*Stat* family, were shared across different HPC types, while each type also displayed unique changes, with corticotropes showing the largest number of DEGs. This is consistent with the function of corticotropes as a key immunomodulator under systemic inflammation [5].

**Fig 1:**
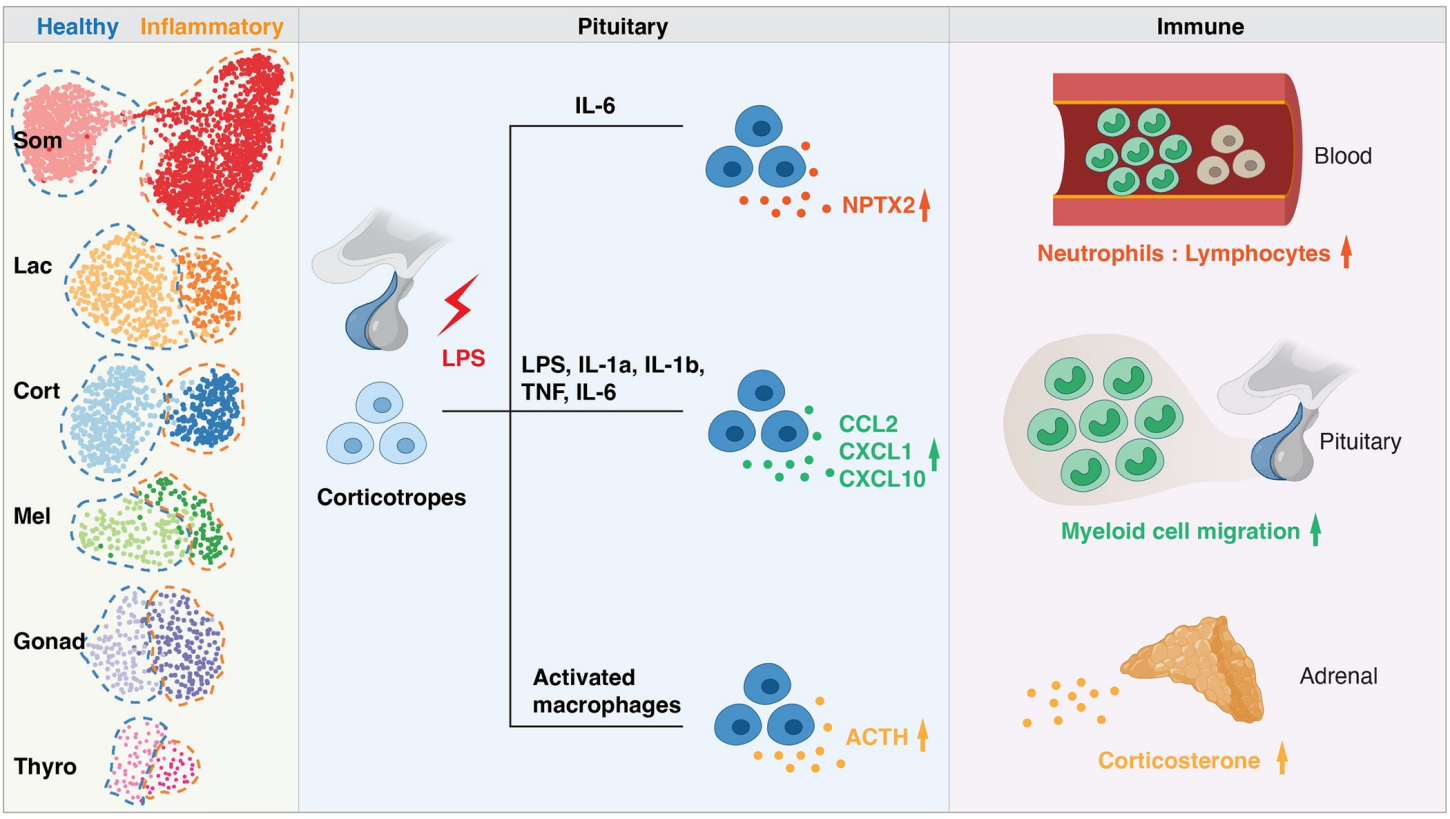
Pituitary-immune interactions under systemic inflammation. Left panel: single-cell RNA sequencing reveals state changes in 6 types of pituitary hormone-producing cells. Som, somatotropes; Lac, lactotropes; Cort, corticotropes; Mel, melanotropes; Gonad, gonadotropes; Thyro, thyrotropes. Middle panel: corticotropes display the strongest reaction upon LPS stimulation. Secretion of classical hormone ACTH (adrenocorticotropic hormone) and nonconventional bioactive molecules, such as the neuronal pentraxin NPTX2 and cytokines/chemokines (e.g., CCL2, CXCL1, CXCL10), is increased, which is regulated by different cellular and molecular signals. Right panel: these secreted proteins from corticotropes modulate immune functions in the periphery, by increasing neutrophil-to-lymphocyte ratios in the blood, promoting myeloid cell migration to the pituitary, and triggering corticosterone release in the adrenal gland. Changes in the immune compartment may in turn regulate pituitary function and physiology. Created with BioRender.com.

Do HPCs secrete nonconventional bioactive factors other than hormones to regulate immune response? To address this question, the authors mined the sequencing datasets and identified 13 candidate genes encoding secreted proteins that were up-regulated in at least 1 HPC type. Among these genes, *Cartpt* and *Nptx2* were specifically up-regulated in the corticotropes of the pituitary but not in other brain regions or peripheral organs after LPS treatment. NPTX2 is a neuronal pentraxin, implicated in synaptic plasticity and excitatory synaptogenesis [6,7]. It has recently been shown that NPTX2 interacts with the complement protein C1q to restrict microglia complement-dependent synapse engulfment in neurodegeneration [8]. Despite the pentraxin superfamily being known for its evolutionarily conserved roles in innate immunity [9], the function of *Nptx2* in the context of inflammation has not been studied. Through tissue-specific manipulation of *Nptx2*, the authors demonstrated that the pituitary was a major source of NPTX2 in the serum, and the increased NPTX2 secretion by the pituitary affected immune balance by regulating the blood neutrophil-to-lymphocyte ratio. Intriguingly, *Nptx2* up-regulation in corticotropes seemed to depend on IL-6 but not other cytokines tested.

In addition to novel bioactive agents such as NPTX2, certain cytokines and chemokines (e.g., CCL2, CXCL1, CXCL10) were also elevated in HPCs. This was likely a direct response of HPCs to proinflammatory cytokines during inflammation. These observations underscore the possibility of direct communications between HPCs and peripheral immune cells. Indeed, cell lines modeling corticotropes were able to promote myeloid cell migration in vitro. Consistently, the numbers of neutrophils and recruited macrophages, which express receptors sensitive to those cytokines/chemokines, were increased in the pituitary after LPS treatment.

Building upon these results, the authors decided to characterize the function of myeloid cells, particularly recruited macrophages, for their potential role in regulating HPC hormonal secretion. They found that LPS-treated macrophages were sufficient to boost ACTH-related gene expression and its secretion in corticotropes in vitro, pointing to an immunomodulatory mechanism. Furthermore, macrophage/microglia depletion reduced serum ACTH and corticosterone, while *Ccl2* overexpression increased their levels. These data suggest that recruited macrophages in the pituitary may regulate HPC activities independently of hypothalamus-derived hormone regulation under inflammatory conditions.

In this study, Yan and colleagues have provided a valuable resource for future investigations into pituitary function and physiology in both health and during immune challenges. While the focus was on the effects of LPS treatment, these datasets also involved other models, such as cecal ligation and puncture, polycytidylic acid and tumor necrosis factor α injections, enabling more detailed comparisons between stimuli. The authors defined the cell type-specific state shifts of HPCs at the transcriptomic level, offering an unbiased view of the inflammation-induced gene expression changes in the pituitary, a critical hub along the HPA axis. By characterizing nonconventional secreted molecules by HPCs, they provided new perspectives on the immunomodulatory function of pituitary cells, moving beyond the hormone-centric dogma and demonstrating novel modes of bidirectional communication between HPCs and immune cells during inflammation (**Fig 1**).

These exciting discoveries also raise numerous questions for future studies. For example, while this paper mainly centered on corticotropes, given that other HPC populations also up-regulate cytokines/chemokines and express distinct sets of secreted proteins, what are the roles of those cells? The observation of NPTX2 affecting peripheral immune balance is promising, but how is this achieved, and does it have other functions? What about the rest of the secreted proteins? HPCs and immune cells communicate via cytokines and chemokines, indicating a potential loop. How is this process initiated? How do different populations of immune cells and HPCs coordinate their responses over time? As microglia also secrete and respond to immune stimuli, do they have distinct functions compared to macrophages? How is this regulatory mode integrated with the classical hypothalamus control of HPCs in hormone release? Lastly, since hormonal, cytokine/chemokine, and nonconventional secreted molecules represent distinct mechanisms of immune modulation, how do they cooperate to finetune a proper immune response? Addressing these questions will also shed light on pituitary-immune interactions in many other contexts.
